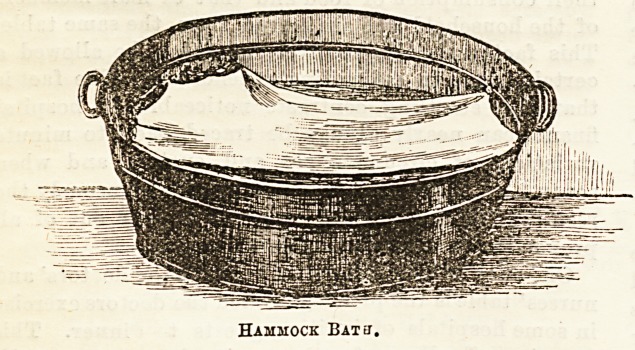# Practical Departments

**Published:** 1895-11-23

**Authors:** 


### PRACTICAL DEPARTMENTS.

### A NEW NURSERY BATH.

The Hammock Bath (Spark's patent) made by Messrs. O'Brien, Thomas, and Co., is likely to obtain much favour in the nursery. The hammock, it will be seen by the accompanying drawing, is slung by strong cord to the sides of an ordinary bath; it is made of soft material, on which baby may happily repose while the hands of nurse or mother are both left free to carry on the washing process with ease and comfort. To raise or lower the hammock adjustment of the cord is all that is necessary, and by unfastening the hooks at

Hammock Bath.

the foot and allowing the child to slip right down into the water, "splashing," the delight of every child's bath, can be freely enjoyed, while the upper part of the hammock keeps head and shoulders well supported out of harm's way. In cases of convulsion or of spinal disease it can be readily understood that this contrivance may be of great assistance, and in giving medicated baths, allowing as it does "access of the water to every part of the child's body, which meanwhile lies at ease in perfect security." Nurses who have tried the experiment speak most approvingly of the device, which will, no doubt, become widely popular as it is better known.

## Figures and Tables

**Figure f1:**